# A Sensor-Based Screening Tool for Identifying High Pelvic Mobility in Patients Due to Undergo Total Hip Arthroplasty

**DOI:** 10.3390/s20216182

**Published:** 2020-10-30

**Authors:** Xueyang Wang, Arham Qureshi, Abhinav Vepa, Usama Rahman, Arnab Palit, Mark A. Williams, Richard King, Mark T. Elliott

**Affiliations:** 1 WMG, University of Warwick, Coventry CV4 7AL, UK; xw839.sun@gmail.com (X.W.); A.Palit.1@warwick.ac.uk (A.P.); M.A.Williams.1@warwick.ac.uk (M.A.W.); 2 University Hospitals Coventry & Warwickshire NHS Trust, Coventry CV2 2DX, UK; Arham.Qureshi@uhcw.nhs.uk (A.Q.); Vepa.Abhinav@nhs.net (A.V.); Usama.Rahman@nhs.net (U.R.); Richard.King@uhcw.nhs.uk (R.K.)

**Keywords:** total hip arthroplasty, inertial measurement unit, pelvic tilt, wearables

## Abstract

There is increasing evidence that pelvic mobility is a critical factor to consider in implant alignment during total hip arthroplasty (THA). Here, we test the feasibility of using an inertial sensor fitted across the sacrum to measure change in pelvic tilt, and hence screen for patients with high pelvic mobility. Patients (*n* = 32, mean age: 57.4 years) due to receive THA surgery participated in the study. Measures of pelvic tilt were captured simultaneously using the device and radiograph in three functional positions: Standing, flexed-seated, and step-up. We found a strong correlation between the device and radiograph measures for the change in pelvic tilt measure from standing to flexed-seated position (R^2^ = 0.911); 75% of absolute errors were under 5 degrees. We demonstrated that the device can be used as a screening tool to rapidly identify patients who would benefit from more detailed surgical planning of implant positioning to reduce future risks of impingement and dislocation.

## 1. Introduction

Hip dislocation is among the most prevalent complications of hemiarthroplasty (HA) and total hip arthroplasty (THA), with an incidence of up to 10% following primary THA, and 28% following revision THA [1]. Economic analyses have estimated a large cost-burden regarding the management of post-operative hip dislocations, with estimates suggesting that early post-operative hip dislocations increase the overall costs of HA, primary THA, and revision THA by 472%, 342%, and 352%, respectively [2]. It has been well established that one of the most critical etiological factors precipitating post-operative hip dislocations is the intra-operative positioning of the acetabular cup prosthesis component. Historically, Lewinnek et al. defined a “safe zone” of 40 ± 10° inclination and 15 ± 10° anteversion for acetabular cup positioning in order to reduce the risk of hip dislocation [3]. However, a recent systematic review has concluded that the placement of acetabular cups within the safe zone does not confer any protection against the post-operative risk of hip dislocation [4].

Emerging evidence now indicates that, due to the biomechanical relationships between spinal and pelvic mobility [5], some patients may exhibit varying pelvic tilt depending on the functional hip position, which may (along with other factors) contribute to acetabular anteversion, and the consequent elevated risk of post-operative hip dislocation despite acetabular cup component positioning within the Lewinnek safe zone [6,7,8]. In an analysis of over 1500 patients, with a 40° inclination and 20° anteversion in acetabular cup fitting, 17% of patients were expected to have a level of pelvic mobility that would result in a functionally mal-orientated acetabular position. Furthermore, by factoring in a ±5° surgical positioning error, this increased as high as 51% [9]. Based on this rationale, research has now focused on exploring the relationship between functional pelvic tilt and post-operative hip dislocation [8], with a view to reducing the incidence, and subsequent cost-burden, of this complication.

Measuring the functional variation in pelvic tilt remains a challenge. Assessments can be made during surgery [10,11,12], but this, by nature, is invasive and requires physical repositioning of the patient, often prolonging total time under anaesthetic. Moreover, only a limited assessment of pelvic motion can be achieved whilst patients are in the supine or lateral positions during surgery. This has led to the notion of “functional” pre-operative radiographic imaging, which involves the measurement of pelvic tilt in different purposeful hip positions (e.g., sitting, standing), in order to optimize intra-operative acetabular cup positioning and, thus, reduce post-operative prosthetic hip dislocation rates [5,9,13,14,15]. While this current state-of-the-art approach delivers a personalized surgical plan to patients undergoing THA [16,17], it is resource intensive, requiring additional radiology time and a detailed analysis of the resulting radiographs. Therefore, an efficient, rapid method is required to screen for patients with high pelvic mobility. Those identified with high pelvic mobility can proceed to receive detailed pre-surgical planning to minimize the risk of acetabular cup malorientation, while those with low pelvic mobility can receive standard acetabular positioning with limited risk of complications.

Here, we have conducted an observational study, for the first time, aiming to investigate the feasibility of using inertial measurement units (IMUs) as a point-of-care screening tool, in order to risk-stratify patients based on their pelvic mobility in the pre-operative clinic setting. A number of studies have investigated the measurement of pelvic tilt using sensor devices. One earlier study investigated the measurement in hockey players [18]. This was achieved using an electrical device that consisted of an electrocardiogram electrode, metal sheet, small light, and battery, and was validated against radiographic measurements. The device was developed only for a specific test, and was therefore not suitable for wider applications. More recently, an IMU-based pelvic tilt measurement method was developed for the measurement of runners during indoor sprint activities [19]. The IMU was attached to the body with double-sided tape above the participants’ L5 spinous process, overlapped with a plastic surgical tape, and covered with form-fitting sprint pants. However, this strong adhesive attachment could result in increased skin movement artefacts.

In this study, we used a bespoke sacral clamp to measure the change in pelvic tilt in a patient. The device’s accuracy was evaluated against plain-film radiograph measurements of pelvic tilt captured at the same time in different functional hip positions [20]. We further investigated if the patients’ gender or body mass index (BMI) impacted on the level of error observed, hypothesizing that those with a higher BMI would have increased skin movement artefacts that reduced the accuracy in tracking the pelvis.

## 2. Materials and Methods

### 2.1. Pelvis Motion Tracking Device

**Figure 1:**
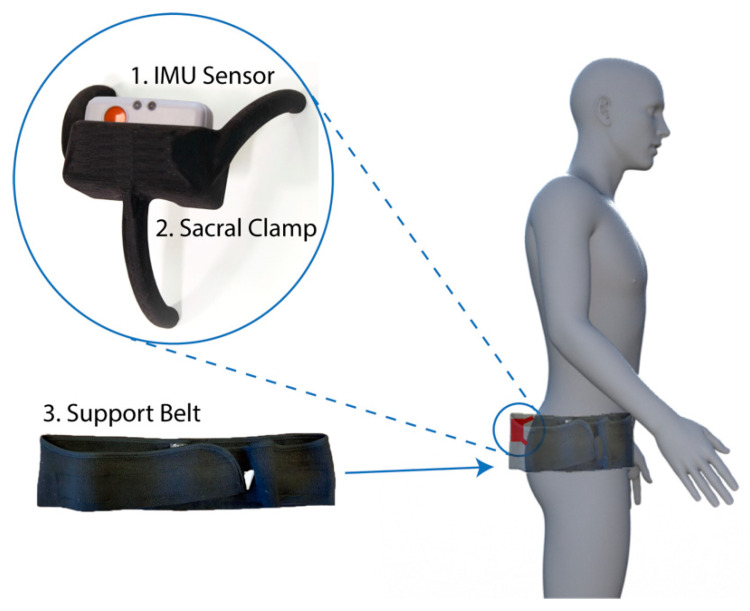
Device components and fitting location. The device consisted of three components: Inertial measurement unit (IMU) sensor (1), a bespoke sacral clamp (2), and support belt (3). The device was attached across the sacrum and held in place by the belt.

The portable device developed to track the motion of the pelvis consisted of three main components (Figure 1):

1. Inertial measurement unit (IMU). A research-grade IMU (Shimmer3; Shimmer, Dublin, Ireland) was used to calculate the pelvic tilt. The device (dimensions, 51 × 34 × 14 mm) housed three sensors: An accelerometer, gyroscope, and magnetometer, with the respective measures recorded in 3-axes. For this study, we only used the data from the accelerometer (sensitivity: 660 ± 19.8 mV/g). The device connected to a host computer wirelessly via a Bluetooth serial connection. For these experiments, a script was produced using the Matlab programming language (v2017b; Mathworks Inc., MA, USA) to capture the data with a sampling rate of 200 Hz.
2. Sacral clamp. The IMU device was housed in a custom-designed “sacral clamp” which allowed the sensor to lie across the participant’s sacrum. The sacral area was chosen due to it having the least amount of skin/fat thickness between the sensor and the pelvic bone and hence provided a feasible location to best track the tilt of the pelvis accurately.
3. Support belt. To hold the sacral clamp securely in place, a wide elasticated belt was fitted around the individual’s waist. The belt used was a pregnancy support belt, made of elasticated material, such that it provided the flexibility for the individual to move freely, while holding the sacral clamp firmly in place.

The clamp was designed such that it was not adhered to the skin (which would result in measurements of skin movement artefacts rather than pelvic tilt). Rather, the aim was for the clamp to track the movement and tilt of the sacrum as closely as possible to determine the change in pelvic orientation between positions. The elasticated belt provided enough tension to hold the clamp against the body, whilst allowing it to move with the sacrum.

### 2.2. Sample

Patients were recruited through the University Hospitals Coventry and Warwickshire National Health Service (NHS) Trust as part of the evaluation of X-ray, acetabular guides, and CT in THR (EXACT) trial [20]. Participants from both the control and intervention arms of the trial were recruited during the period between November 2017 and November 2018, following the inclusion and exclusion criteria stated in the Supplementary Materials, Table S1. As part of the trial, they were provided with an information leaflet and gave written consent prior to participating. Upon attending the hospital, participants were requested to wear the device during the acquisition of their pre-operative radiographs. Data from a total of *n* = 32 patients (mean age: 57.4 ± 9.4 years, female: *n* = 17) was successfully collected during the trial period (see Table S2).

### 2.3. Ethical Review

This study formed part of the EXACT clinical trial; the protocol was approved by the West Midlands-Solihull NHS Research Ethics Committee (Ref: 17/WM/0261).

### 2.4. Procedure

Patients were dressed in a hospital gown, proceeded to the radiography room, and were shown how and where the IMU, sacral clamp, and elastic belt would be fitted. Patients were able to decline wearing the device and just proceed with the radiography if they wished.

Patients first received a standing anterior radiograph, prior to the IMU being fitted. The IMU was placed into the slot in the sacral clamp such that it was firmly secured and tracked the tilt of the clamp. The clamp was then placed over the patient’s sacrum, with the upper 2 “legs” of the clamp located over the sacroiliac joints (SIJs). Where the SIJ could not be easily palpated, the appropriate position of the clamp was estimated by palpating the anterior superior iliac spines and subsequently placing the upper 2 “legs” at this height over the patient’s lower back. This was then held in place by tightly fastening the elasticated belt around the patient’s pelvis, and fastened securely with two Velcro straps on each side. IMU data recording was started and stopped using the custom Matlab software, which linked to the IMU via a Bluetooth connection.

**Figure 2:**
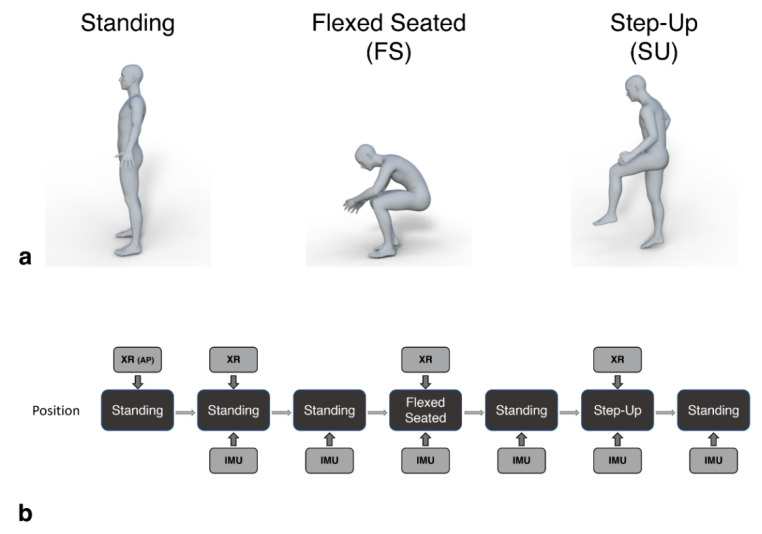
Patients assumed standing, flexed-seated, and step-up positions during the procedure (**a**). These positions were captured by the IMU device in parallel with lateral lumbar spine radiographs (XR), as shown in (**b**). An initial anterior pelvic radiograph was captured at the start (XR (AP)). Patients held each position for a minimum of 5 s, during which the image was taken and the IMU three-dimensional accelerations were recorded. Additional standing positions were recorded by the IMU to provide a baseline tilt measure before each radiograph.

Pelvic tilt was measured whilst the patient was asked to adopt three positions: Standing, flex-seated (FS) and step-up (SU) positions (Figure 2a). These are positions which occur often in daily activities and are likely to result in the highest angles of hip flexion in which pelvic tilt should be measured [9]. Further, this protocol is the current state-of-the-art for assessment of pelvic mobility and pre-surgical planning of implant position, using radiograph measurements [20,21]. By using the same measures, we were able to do a direct comparison of our device measurements compared to the radiograph measures.

Lateral lumbar spine radiographs were captured in parallel with IMU measures for each position. Additional baseline standing measures were recorded by the IMU in between each position captured by the radiography (Figure 2b). Patients were required to maintain each position for a minimum of five seconds, during which the IMU data and radiographic image were captured. The time of the radiograph relative to the start time of the recording of the IMU data was noted to allow the IMU measured tilt in that period to be directly compared to that calculated from the images.

At the end of each capture, the three-dimensional acceleration data captured by the IMU was saved and labeled. The radiographic images for each captured position were assessed independently by staff at Corin Group (Gloucestershire, UK) as part of their optimized positioning system assessment [22]. The IMU tilt angle measures were calculated by a researcher at the University of Warwick. Once the IMU measures had been recorded, the radiograph measures were sent to the researcher for comparison.

As a screening tool, we were interested in the change of pelvic tilt between positions, rather than the specific values of tilt in each position. This approach further avoided any complex calibration procedure that would be otherwise required to align the IMU axes with the pelvis axes. Hence, we used the standing position as a baseline measure of tilt and calculated the change in pelvic tilt when patients assumed the FS and SU positions, relative to the baseline measure. The same calculation was made based on the measures collected from the radiographs, allowing a direct comparison to the IMU measures.

### 2.5. IMU Pelvic Tilt Measure

The data from the 3-axis accelerometer built into the IMU was used to calculate pitch and roll values whilst participants assumed the different postural positions.

**Figure 3:**
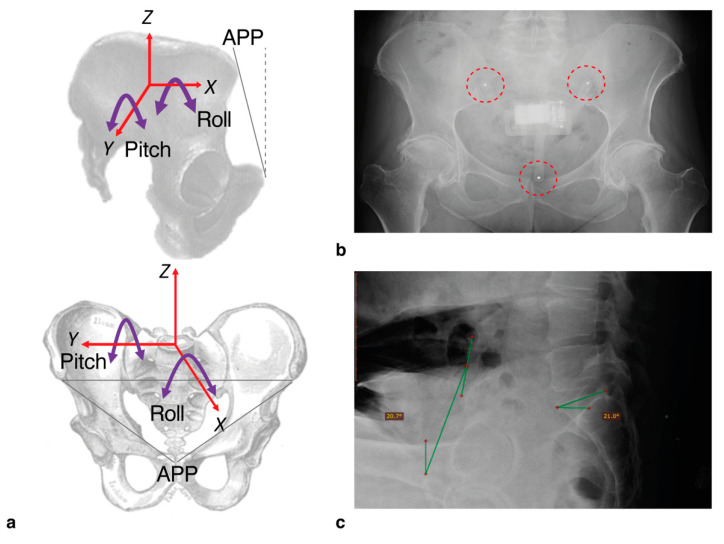
With the IMU device in place, we were able to measure pelvic tilt according to the axes shown (**a**). For this study, we only measured pitch (i.e., tilt in the anterior-posterior direction, around the *Y*-axis). For the radiograph assessments, the tilt was measured according to the angle of the anterior pelvic plane (APP) defined by the plane between the anterior superior iliac spines and the pubic tubercles, relative to the vertical (dashed line). To check positioning of the device following capture, 3 mm steel bearings were inserted into the legs which appeared clearly on radiographs ((**b**); highlighted by dashed circles). An example radiograph showing the annotated measure of tilt based on APP relative to vertical (green lines) is shown in (**c**). Sacral slope was also calculated but not used in this study.

In the main study, the patients adopted the requested position and were requested to remain still for the short period of data capture (i.e., 5 s), during which time the radiograph was also captured. By remaining still, we can assume that only a gravity component was acting upon the accelerometer, allowing the calculation of the pitch (*θ*; Equation (1)) and roll (*φ*; Equation (2)) measures of pelvic tilt using the accelerations (*A*) measured in 3-dimensions [23]:

(1) $$\theta =\mathrm{atan}\left(\frac{-A_{x}}{\sqrt{A_{y}^{2}+A_{z}^{2}}}\right)$$

(2) $$\varphi =\mathrm{atan}2\left(A_{y},A_{z}\right)$$

where atan is the inverse tangent, and atan2 is the four-quadrant inverse tangent. Pitch is defined as the estimated angle of pelvic tilt around the frontal axis, *Y* (Figure 3a), in the anterior-posterior (AP) direction, and roll is the angle estimated around the sagittal axis, *X* (Figure 3a), in the medial-lateral direction (ML). In this study, we focused only on the pitch of the pelvis, due to the two-dimensional radiograph images only providing measures in this axis for comparison.

Before collecting patient measurements, we did a number of pre-tests and design iterations of the IMU and clamp. This included calibrating and assessing the accuracy of the IMU, achieved by tilting the device through a range of angles between −30 and +30 degrees using an industrial robot arm (KUKA KR10 R900, KUKA AG, Augsburg, Germany). Using the recorded values, pitch was calculated using Equation (1). Mean absolute error at ±30 degrees tilt was recorded as 0.41 ± 0.32 degrees (see Appendix A for full details).

To tackle the potential errors introduced by patients’ swaying and making unrelated movements during capture, the most stable 300 ms segment during the capture period was selected by finding the window with the lowest standard deviation after applying a zero-phase moving average filter with a hop size of 0.1 s. This ensured the maximum accuracy of pitch measurements synchronous with the radiographic measure. Finally, to ensure that the clamp was positioned correctly in place during capture, spherical steel bearings (3 mm diameter) were inserted into the ends of the three “legs” of the clamp. These appeared clearly on the radiograph allowing a visual assessment of the clamp position (Figure 3b).

### 2.6. Radiographic Measures of Pelvic Tilt

Pelvic tilt was measured from a lateral pelvic radiograph, captured while the patient assumed each of the three positions: Standing, FS, and SU. On each image, the anterior pelvic plane (APP) was defined by points at the anterior superior iliac spines and the pubic tubercles (Figure 3a). Pelvic tilt was subsequently calculated by measuring the angle between the APP and the vertical (Figure 3c [22,24]). Change in pelvic tilt was recorded for the FS and SU positions by calculating the difference between the tilt measured in these positions relative to the standing measure. Positive values represented an increase in anterior pelvic tilt.

### 2.7. Analyses

Anterior pelvic tilt change for SU and FS positions relative to standing were calculated from the IMU data and subsequently compared to the same measures derived from the radiographic images. The ability of the IMU device to track the pelvic tilt was determined using a Spearman correlation, for both FS and SU measurements.

The measurement errors between the device and the radiograph measurements were assessed using Bland-Altman plots [25]. Accuracy was further investigated through the analysis of the absolute errors, defined as the unsigned difference between IMU and radiograph measures. These were then compared to body mass index, using a Pearson correlation and tested for differences between genders using a mixed-ANOVA (position (FS, SU) × gender (female, male)).

Finally, as a potential screening tool, we considered the accuracy of classifying patients into those with and without high levels of pelvic tilt using the FS data (defined as ≥13 degrees of rotation when moving from standing to the FS position [9,26]). Based on this, we calculated the sensitivity (Equation (3)), specificity (Equation (4)), and accuracy (Equation (5)) of the device, where *TP* was the number of true positive results; *TN*, the number of true negatives; *FP*, the number of false positives; and *FN*, the number of false negatives:

(3) $$Sensitivity=\frac{TP}{TP+FN}$$

(4) $$Specificity=\frac{TN}{TN+FP}$$

(5) $$Accuracy=\frac{TP+TN}{TP+TN+FP+FN}$$

## 3. Results

### 3.1. Correlation with Radiograph Measures

**Figure 4:**
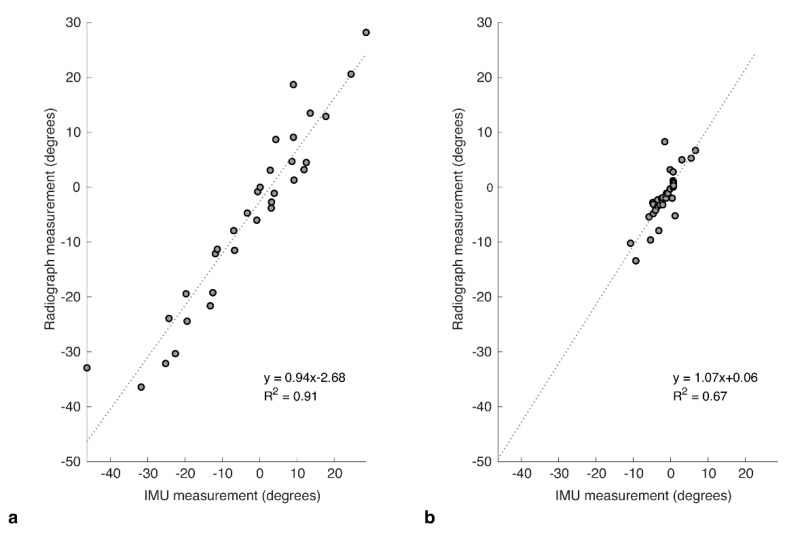
Correlation plots for IMU against the corresponding measures taken from radiograph images captured at the same time for relative change in pelvic tilt in (**a**) flexed-seated (FS) and (**b**) step-up (SU) positions, relative to standing.

There was a difference in the level of correlation for the two functional positions, with FS showing a strong correlation between the IMU and radiograph measures (R^2^ = 0.911, *p* < 0.001; Figure 4a). SU showed a more moderate correlation (R^2^ = 0.673, *p* < 0.001; Figure 4b).

### 3.2. Bland-Altman Analysis

**Figure 5:**
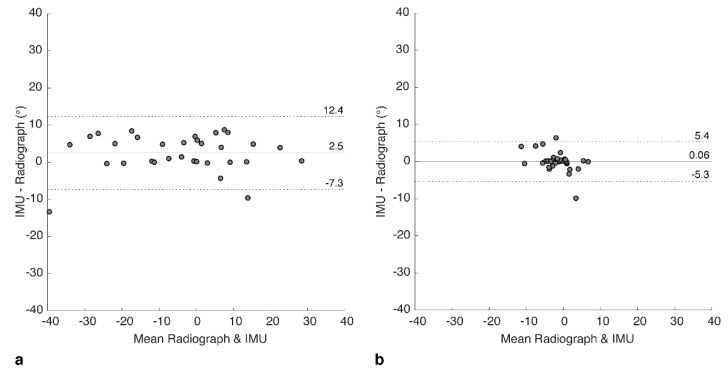
Bland-Altman plots showing difference between IMU and radiograph measures for (**a**) flexed-seated and (**b**) step-up positions. Solid horizontal line indicates bias, dashed lines indicate upper and lower limits of agreement (95% confidence intervals).

The agreement between IMU and radiograph measures was explored further using Bland–Altman analyses to investigate the bias and variance for each of the two positions. For FS, the mean of differences (bias) between the two measures was 2.53 ± 5.02 degrees (Figure 5a), showing that the IMU tended to over-estimate the tilt. An upper limit of agreement (U-LOA) measured as the 95% confidence interval above the mean was 12.35 degrees, and the lower limit measured (L-LOA) measured as the 95% confidence interval below the mean was −7.32 degrees.

In contrast, for SU, the bias was calculated as 0.06 ± 2.74 degrees (Figure 5b), with a U-LOA of 5.43 and L-LOA of −5.30 degrees. Hence, bias and variance of the errors were both lower for SU but should be considered in the context of a lower range of measured tilt values compared to FS.

### 3.3. Error Distribution

**Figure 6:**
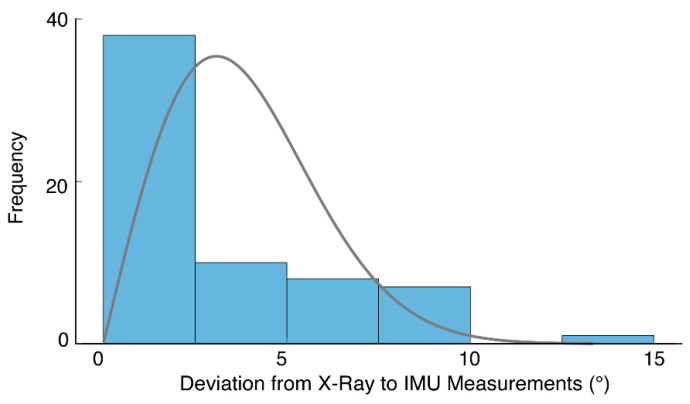
A histogram of absolute errors (shaded bars) based on both FS and SU positions (i.e., 64 measurements in total). The results suggest errors follow a Poisson distribution (solid line), with a skew towards lower values.

Considering the combined dataset again across both SU and FS positions, we analyzed the distribution of absolute errors between IMU and radiograph measures. Errors appeared to follow a Poisson distribution (Figure 6) with a skew towards lower values (median = 1.3°). Seventy-five percent of errors were under 5° (i.e., third quartile = 4.9°).

We subsequently investigated if there was a relationship between the size of the error and the magnitude of the pelvic tilt, which revealed a stronger relationship for the SU position (Pearson’s R = 0.52, *n* = 32, *p* = 0.002) than for the FS position, which was not significantly correlated (Pearson’s R = 0.24, *n* = 32, *p* = 0.178).

### 3.4. Relationship of Error with Body Mass Index and Gender

Mean body mass index (BMI) was 29.2 ± 4.6 kg m^−2^. There was a wide spread of body sizes in the sample, with 7 patients (22%) being classified as normal weight (BMI between 18.5 and 24.9), 14 (44%) patients overweight (BMI between 25 and 29.9), and 11 (34%) patients obese (BMI 30 or over); see Table S2 for a full breakdown. Correlation analyses between the absolute error and BMI across all participants showed no relationship for either the FS (Pearson’s R = 0.004, *n* = 32, *p* = 0.982) or SU (Pearson’s R = −0.044, *n* = 32, *p* = 0.811) positions. Similarly, we found no difference in absolute error between male and female participants. Using a mixed-ANOVA (gender × position), we found that the absolute error was significantly larger for FS (M = 4.30 ± 3.62) than SU (M = 1.57 ± 2.26) positions (F(1, 30) = 17.85, *p* < 0.001), which was in agreement with the Bland-Altman results above. There was no significant difference in absolute error between genders overall (F(1,30) = 0.042, *p* = 0.839) or genders within each position (F(1, 30) = 1.049, *p* = 0.314).

### 3.5. Classification Accuracy

Using the radiograph measures as ground-truth values, we generated a contingency table (see Table 1) which shows the performance of our device based on the FS results. From Equations (3)–(5), we calculated the Sensitivity: 84.6%, Specificity: 95.0%, and Accuracy: 90.9%.

## 4. Discussion

The correlations between IMU and radiographic measurements of pelvic tilt were strong for FS and moderate for SU, suggesting that our sacral clamp was effective in tracking pelvic movements. Bias values for both the FS and SU positions were also low for both FS (<3 degrees) and SU (<1 degree) positions. The variability in the errors between the IMU and radiographic measurements was higher in the measures for the FS position than the SU position. Seventy-five percent of the errors across all measurements were within 5 degrees of the radiograph measurements. In the context of screening for high levels of pelvic mobility (defined as ≥13 degree change in pelvic rotation in either anterior or posterior directions), overall accuracy was found to be 90.9%. Therefore, the prototype device performed well, given the challenges of tracking the movement of the pelvis. Other approaches for measuring tilt include basic palpation of anatomical landmarks, but this has only shown accuracy for anterior pelvic tilt [27]. The pelvic goniometer is a manual instrument that can also be used to measure pelvic tilt and research has indicated that they can do so quite accurately. However, they can often require a lot of time and training to use and are less commonly used in current practice [28]. Other techniques have been described in the literature using calipers or markers, however these have not been used widely in clinical practice, due to difficulty in standardization and thus unreliability [29]. While there have been studies using IMUs to measure pelvic tilt (e.g., [19]), the challenge is to avoid strong adherence to the skin which doesn’t necessarily track the tilt of the pelvis itself. Here, we have shown that measurement of functional pelvic tilt can be achieved using a wearable, portable IMU sensor. Moreover, the use of a custom designed sacral clamp allowed the sensor to move in a way that closely tracked the tilt of the pelvis, as validated through comparison with radiographic measures.

There was no correlation between the amount of pelvic tilt change and the size of the errors for the FS position, which recorded a wide range of measurements (approx. ±30 degrees). This indicated that there was no systematic slippage or tracking error exhibited by the clamp. It is possible that the larger errors could have been caused by the device being knocked, e.g., hitting the seat base or back-rest when in the sitting position. We incorporated metal bearings into the clamp, which allowed a gross assessment of position, and did not highlight any issues, with medical staff fitting the clamp in the correct position. However, it was difficult to specifically quantify any movement of the device between positions. Future revisions of the prototype will focus on design changes to the device (e.g., reduction in the profile of the clamp) and also to the protocol relating to data capture, to minimize risk of unrelated movement (e.g., using a stool where there is a reduced surface area of the seat-base and no backrest).

Since obesity is significantly associated with hip osteoarthritis and thus THA [30], it is likely that a large proportion of patients being assessed using this device would be classified as overweight or obese. We predicted that our device’s accuracy may be reduced in those with higher BMI, due to the increased subcutaneous adiposity between the sacral bone and the device. However, our study population, which represented patients of all BMI categories, showed no correlations between the size of the error and the BMI. Similarly, there were no gender differences related to the size of the error. This suggests that our device is robust against different body sizes and habitus’; however, a larger study is required to validate this fully.

**Figure 7:**
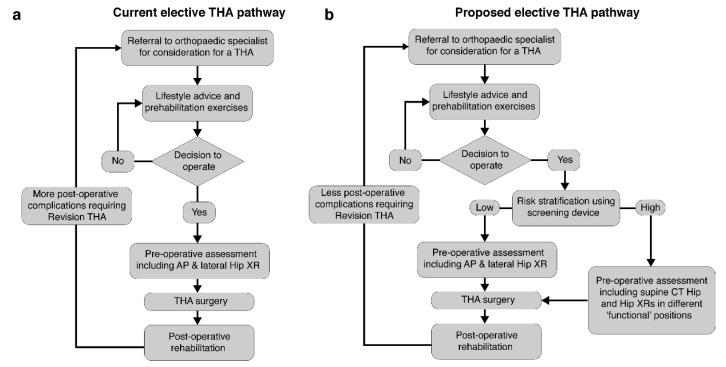
The typical current elective total hip arthroplasty (THA) pathway is shown in (**a**). We propose a revised pathway to include the rapid screening using the device (**b**). Patients identified with a high pelvic mobility received a detailed pre-operative assessment.

Our pelvic tilt measurements were recorded pre-operatively, and we envisage the device to be used as a cost-effective point-of-care screening tool in order to pre-operatively risk-stratify patients awaiting THA. This would allow the more extensive pre-operative radiographic assessments to be reserved for high-risk patients, as shown in the example care pathway in Figure 7. It would further reduce overall costs and radiation exposure in the population of patients awaiting a THA, whilst also maintaining reduced post-operative hip dislocation rates.

## 5. Limitations

There were a number of limitations to the study which should be considered in the interpretation of results. Due to the limited sample size and age range of the participants in this study, we could not generalize these results to the wider population of patients awaiting THA surgery. In particular, the mean age of participants was 57 years; in comparison, the average age recorded by the UK’s National Joint Registry in 2019 was 67.6 years for males and 70.0 years for females [31]. Hence, our sample average age was substantially younger. In particular, one of our participants was 23 years, which reduced the overall mean; however, as they met the original inclusion criteria, it was not appropriate to exclude their data.

Our baseline measure was based on the standing position. A more comprehensive assessment could be achieved by using a baseline measure of pelvic tilt from the supine position, which would further allow a calculation of relative change between supine and standing [9]. This could be achieved by a more low-profile design of the sacral clamp, realized by embedding the sensor electronics into the clamp (rather than using a third-party IMU). However, as an assessment in the supine position is less practical than standing and seated positions, and the range of motion between standing and flexed-seated (FS) is generally larger than supine to standing, we believe it is likely that overall, the standing to FS measure was a better indicator of high pelvic mobility.

Our assessments, along with those captured by radiograph, were based on single measurements for each functional position. In order to reduce the recorded errors further and hence reduce the risk of incorrect screening decisions when using the device, repeated measurements may have been required, and this may direct the focus of future research. This repeated-measure approach can also be used to further understand the levels of variability in an individual’s pelvic tilt angle (for example, is it affected by time of day or prior levels of activity?).

In addition, we may be able to improve the accuracy of the algorithm by using a sensor fusion algorithm to determine orientation using all three IMU sensors (accelerometer, gyroscope, and magnetometer using a Kalman Filter or similar approach, c.f. [32]). This would enable the capture of dynamic measurements of pelvic tilt, in terms of both pitch (anterior–posterior tilt) and roll (medio-lateral tilt), and hence provide a full mapping of pelvic tilt for a wide range of daily activities. This is in contrast to the current use of radiographs, which can only capture a snapshot in time of pelvic mobility; they are not practical to measure the full extent of variability in pelvic movements. These dynamic measures will also be an important contribution to model-based planning of acetabular cup positioning, based on the bone physiology of patients (captured using a computed tomography (CT) scan) and used to identify potential post-operative prosthetic impingement [33,34,35] or bony impingement [36,37]. Using the device with a dynamic tilt measurement algorithm, the pelvic tilt mapping for activities of daily living can be used to validate and further develop the impingement identification models by measuring the actual effect of pelvic tilt on post-operative impingement.

## 6. Conclusions

We have demonstrated the feasibility of using an IMU positioned across the sacrum, using a bespoke clamp design, to accurately measure change in pelvic tilt. Based on the study presented, the device can be used to screen for high pelvic mobility in patients due to have THA, thus reducing the need to use multiple radiographs for all patients. Finally, the applications of our device also extend beyond pre-operative THA planning. Due to the complex interplay between lumbar and pelvic mobility, pelvic movements may also play a key role in chronic back pain; one of the leading causes of disability worldwide [38,39,40], and thus our device may enable identification of potentially pathological pelvic movement patterns which can be targeted via physiotherapy.

## Figures and Tables

**Table 1:** Contingency table of results based on a threshold of ≥13 degrees classifying a patient as having high pelvic mobility when moving from standing to flexed-seated position. Values are counts of participants. Key: True positive (TP), false positive (FP), true negative (TN), false negative (FN).

	IMU ≥ 13 Degrees	IMU < 13 Degrees
Radiograph ≥ 13 degrees	TP = 11	FN = 2
Radiograph ≤ 13 degrees	FP = 1	TN = 19

**Figure A1:**
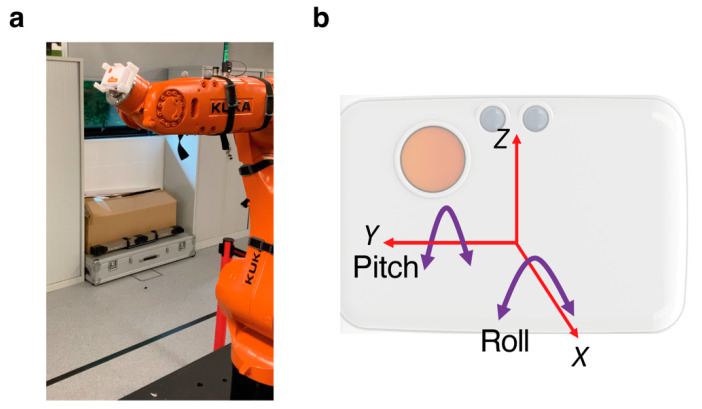
(**a**) Photograph of the equipment set up, with the IMU mounted onto the end-effector of the robot arm. (**b**) Axes of the IMU. Only the pitch was of interest in this study. However, roll was applied to some measurements to check for interactions with the pitch measurements.

**Figure A2:**
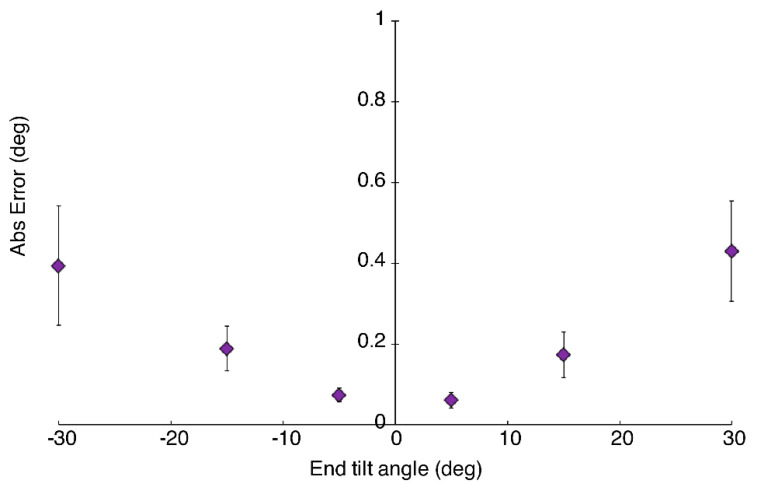
Mean absolute error in pitch measurements for each end-point tilt angle. Error bars represent standard error.

## Supplementary Materials

The following are available online at https://www.mdpi.com/1424-8220/20/21/6182/s1, Table S1: Inclusion and exclusion criteria for patient recruitment onto the trial.; Table S2: Participant information.; Table S3: Start and end pitch/roll for the experiment described in the Appendix. Participant results data is stored at: https://dx.doi.org/10.17605/OSF.IO/UZQ34.

## Appendix A—Measuring the Accuracy of the Sensor Device and Algorithm

Here, we briefly describe the experiment developed to quantify the error in the tilt calculation when compared against a known reference value. This helped us to understand the minimum level of error we could expect to observe due to the sensors themselves. The remaining error could then be associated with the procedure and fitting of the device.

### Appendix A.1. Equipment

1. Inertial measurement unit: A research-grade IMU (Shimmer3; Shimmer, Dublin, Ireland) was used, the same as that used to capture the patient data in our main manuscript. The device (dimensions, 51 × 34 × 14 mm) housed three sensors: Accelerometer, gyroscope, and magnetometer, with the respective measures recorded in 3-axes. For calculating tilt, we only used the data from the accelerometer (sensitivity: 660 ± 19.8 mV/g). The device connected to a host computer wirelessly via a Bluetooth serial connection, with data collected at a sampling rate of 200 Hz, using the Matlab programming language (v2017b; Mathworks Inc., Natick, MA, USA).
2. Robot arm: An industrial robot arm, KUKA KR10 R900 (KUKA AG, Augsburg, Germany), was used to tilt the device in terms of both pitch and roll (see Figure A1a). The robot had a pose repeatability of ±0.03 mm and complied to the ISO9283 standard (https://www.iso.org/standard/22244.html).

### Appendix A.2. Setup

The IMU was mounted firmly in a calibration frame, which was provided with the device. This ensured the device had a flat surface which could be mounted on the robot arm. The device was attached to the robot arm using high quality double-sided tape (as used to attach reflective markers for motion capture applications). It was attached to the end effector of the robot (Figure A1a). This allowed rotation around the *Y*-axis, providing pitch variation, and around the *X*-axis, providing roll (see Figure A1b).

### Appendix A.3. Procedure

The robot moved through a series of pitch and roll movements, with a defined start and end value (see Table S3). In the study, we were only interested in measuring the pitch. However, here we also applied roll to check that a non-zero roll measure would not impact on the pitch errors. Maximum pitch rotations were ±30 degrees, maximum roll rotations were ±15 degrees. For each setting, the robot repeated the movement five times, holding at the start and end points for three seconds and moving between positions at a peak rate of 16 degrees per s.

### Appendix A.4. Analysis

The three-axis accelerometer data was low-pass filtered (sixth order Butterworth, cut-off: 5 Hz) and used to calculate the pitch and roll angles of the sensor, relative to the vertical, using Equations (1) and (2) (see main manuscript). A Matlab script was written to identify the static periods in the sensor signals representing the start and end points of each rotation. Within each static period, the tilt angle recorded by the sensor was averaged over a 300 ms period, centered around the mid-point of the static period. Each trial resulted in five measures of the recorded start and end point angles. The start point measure was subtracted from the adjacent end-point value to get an overall change in tilt angle. The actual change in tilt angle was subtracted from the value to get the measurement error. Errors were converted to absolute values and averaged within the trial and then across all trials with the same change in pitch.

As we were only interested in pitch measures for the application reported in the main manuscript, we focused on these results here.

### Appendix A.5. Results

The mean absolute errors for pitch measurements by the device at each end-point tilt angle are shown in Figure A2. The maximum mean absolute error was found to be at ±30 degrees, with values of 0.43 ± 0.30 and 0.39 ± 0.36 degrees, respectively. Therefore, the combined maximum mean error (averaging across both +30 and −30 degree measurements) was 0.41 ± 0.32 degrees. No correlation was detected between the magnitude of the roll and the errors in the pitch measurement.
