# Supplementary material for: A Sensor-Based Screening Tool for Identifying High Pelvic Mobility in Patients Due to Undergo Total Hip Arthroplasty
Source: Sensors (Basel). 2020 Oct 30;20(21):6182. doi: 10.3390/s20216182 (PMC7663251; doi:10.3390/s20216182)
Supplement: Supplementary file 1 [file sensors-20-06182-s001.pdf]

Table S1. Inclusion and exclusion criteria for patient recruitment onto the trial.

Inclusion Criteria	Exclusion Criteria
All patients undergoing an elective primary unilateral THA under the care of an orthopaedic consultant at UHCW NHS Trust.	Those patients deemed by the treating clinician as unsuitable for an uncemented primary acetabular implant for reasons such as: low demand patient, osteoporosis, significant acetabular bone loss.
Provision of written informed consent.	Patients with significant orthopaedic deformities (e.g., fused knee, hip or ankle).
Male or Female aged 18-70 years.	Unable to undergo planning imaging (unable to stand or sit for radiographic imaging, or to lie in a CT scanner).
	Patients currently receiving ionising radiation treatment or scans for other medical conditions.
Able and willing to comply with all study requirements.	Previous entry in the trial (contralateral THA).
	Participation in a clinical trial of an investigational medicinal product in the last 90 days.

Table S2. Participant information.

Participant No.	Gender	Age (years)	Body Mass Index (kg m^{-2})
1	F	54	39.7
2	M	65	30.3
3	F	55	29.3
4	F	70	22.3
5	M	63	29.3
6	F	54	24.8
7	M	58	35.2
8	M	53	36.3
9	F	60	32.7
10	M	47	30.2
11	M	23	24.5
12	F	51	35.6
13	M	58	26.0
14	F	60	29.0
15	F	55	26.7
16	F	59	27.4
17	F	50	27.2
18	F	61	29.7
19	M	51	33.0
20	F	70	24.8
21	F	57	35.6
22	F	49	21.9
23	M	45	27.3
24	M	60	22.9
25	F	70	26.3
26	M	56	28.8
27	M	69	27.5
28	M	63	35.8
29	F	56	34.9
30	F	56	28.0
31	M	70	27.2
32	M	68	24.5

Table S3. Start and end pitch/roll for the experiment described in the Appendix.

Trial No.	Start pitch (deg)	Start roll (deg)	End pitch (deg)	End roll (deg)
1	0	0	5	0
2	0	0	15	0
3	0	0	30	0
4	0	0	-5	0
5	0	0	-15	0
6	0	0	-30	0
7	5	0	10	0
8	5	0	20	0
9	-5	0	-10	0
10	-5	0	-20	0
11	0	0	5	10
12	0	0	15	10
13	0	0	30	10
14	0	0	-5	10
15	0	0	-15	10
16	0	0	-30	10
17	0	0	5	-10
18	0	0	15	-10
19	0	0	30	-10
20	0	0	-5	-10
21	0	0	-15	-10
22	0	0	-30	-10
23	0	5	5	15
24	0	5	15	15
25	0	5	30	15
26	0	5	-5	15
27	0	5	-15	15
28	0	5	-30	15
29	0	-5	5	-15
30	0	-5	15	-15
31	0	-5	30	-15
32	0	-5	-5	-15
33	0	-5	-15	-15
34	0	-5	-30	-15
